# Interventions for enhancing an online infection control course for Latin American countries

**DOI:** 10.1186/2047-2994-4-S1-P254

**Published:** 2015-06-16

**Authors:** M Gonzalez, M Homsi, G Relyea, M Caniza

**Affiliations:** 1 Infectious Diseases, St. Jude Children’s Hospital, Memphis, USA; 2 School of Public Health, University of Memphis, Memphis, USA; 3 International Outreach Program, St. Jude Children’s Hospital, Memphis, USA

## Introduction

In 2010, St Jude began a 12-week infection control (IC) course using e-learning & face-to-face methodologies to train Latin-American infection preventionists in IC competencies. In 2014, we added a weekly hands-on practicum for reinforcing learned course elements. It consisted of using the Infection Control Assessment Tool (ICAT) developed by USAID and Management Sciences for Health, and the WHO Hand Hygiene Self-Assessment Framework (HHSAF); these validated tools aligned with course competencies.

## Objectives

We aimed (1) to compare the knowledge gain among participants with hands-on practicum vs. control, (2) to assess 6-month implementation of quality improvement (QI) projects based on ICAT/HHSAF results, and (3) to determine user acceptance of the electronic format of ICAT/HHSAF tools.

## Methods

We assigned 43 students to an intervention (IC 2014) or control group (IC 2013) and measured knowledge gain over time (pre/post-test); this constituted a 2-group by 2-within repeated measure design. In a 6-month follow-up of the intervention group, participants were asked to use our ICAT/HHSAF electronic format and complete an evaluation.

## Results

Using a two factor repeated measures design we found a significant interaction between the course and knowledge gain over time, F(1,41)= 6.07, p = .018. Knowledge gain was 31% among the intervention group (n=25, p <.001) and 21% among the control group (n=18, p <.001). Exam scores were not different between the courses at pre-test, t(41)=-1.295, p=.968. Follow-up of QI projects indicates that 67% of respondents implemented QI projects based on tool results and 67% have used at least one of the tools again (n=6). Additionally, respondents found the ICAT/HHSAF electronic format to be more attractive (100%), reduce error (67%), and provide improved understanding of results (100%).

## Conclusion

Incorporating hands-on applications to online IC courses can enhance participant learning outcomes. Validated tools such as the ICAT and the HHSAF are feasible options. Interventions for adoption of systematic use of IC needs assessment tools among preventionists in Latin America are needed.

## Disclosure of interest

None declared.

